# Diversity and Functions of Yeast Communities Associated with Insects

**DOI:** 10.3390/microorganisms9081552

**Published:** 2021-07-21

**Authors:** Simon Malassigné, Guillaume Minard, Laurent Vallon, Edwige Martin, Claire Valiente Moro, Patricia Luis

**Affiliations:** Univ Lyon, Université Claude Bernard Lyon 1, CNRS, INRAE, VetAgro Sup, UMR Ecologie Microbienne, F-69622 Villeurbanne, France; simon.malassigne@etu.univ-lyon1.fr (S.M.); guillaume.minard@univ-lyon1.fr (G.M.); laurent.vallon@univ-lyon1.fr (L.V.); edwige.martin@univ-lyon1.fr (E.M.); claire.valiente-moro@univ-lyon1.fr (C.V.M.)

**Keywords:** insect-microbiota interactions, mycobiota, yeast communities, insects

## Abstract

Following the concept of the holobiont, insect-microbiota interactions play an important role in insect biology. Many examples of host-associated microorganisms have been reported to drastically influence insect biological processes such as development, physiology, nutrition, survival, immunity, or even vector competence. While a huge number of studies on insect-associated microbiota have focused on bacteria, other microbial partners including fungi have been comparatively neglected. Yeasts, which establish mostly commensal or symbiotic relationships with their host, can dominate the mycobiota of certain insects. This review presents key advances and progress in the research field highlighting the diversity of yeast communities associated with insects, as well as their impact on insect life-history traits, immunity, and behavior.

## 1. Introduction

With nearly one million described species and 5.5 million estimated ones, insects represent more than 80% of the animal biodiversity on Earth [1]. Such diversity is reflected by a broad spectrum of evolutionary acquired traits, some of them being linked to their feeding mode [2]. The evolutionary success of many insects is closely tied to symbiotic associations with microorganisms having complementary potential that is otherwise lacking in insects and restricts them when inhabiting an ecologically challenging niche or invading new environments [3,4]. Therefore, our understanding of insect biology is facing a paradigm shift where these higher organisms can no longer be considered as an isolated entity and instead should be studied in relation with its microbiota (bacteria, fungi, protists, and viruses) with which it interacts and forms a metaorganism, often referred to as the holobiont [5,6,7,8].

**Figure 1:**
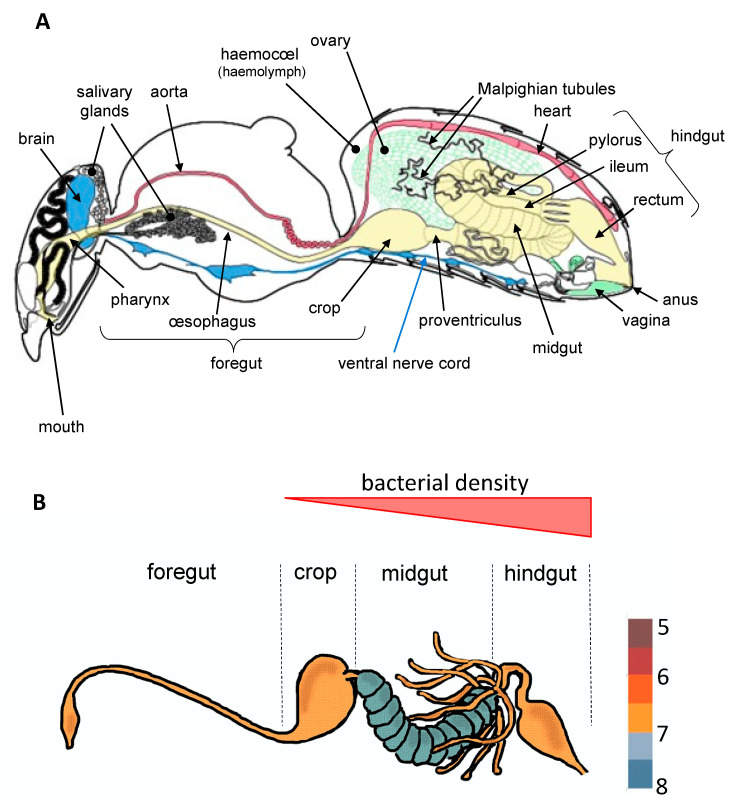
The internal anatomy of an insect (**A**) and variability of bacterial density across the digestive tract (**B**), taking the bee as example (according to Tofilski A.; http://honeybee.drawwing.org, accessed on 5 March 2021 and Kešnerová et al. [14]). All insects present an internal cavity (the hemocoel) containing a circulatory fluid (hemolymph) and all organs forming the digestive (in yellow), reproductive (in green), circulatory (in red), respiratory or nervous (in blue) systems.

To date, most studies have mainly focused on bacteria which establish parasitic, commensal, or symbiotic relationships with their hosts by colonizing different tissues such as ovaries [9], cuticle [10], or specialized host cells (bacteriocytes) often grouped into an organ called the bacteriome [11]. However, most of bacterial microbiota inhabit the digestive tract [3,4], which is composed of three regions with specific functions (Figure 1). These regions vary extensively in terms of morphology and physicochemical properties across insect orders, factors that are known to greatly influence microbial community structure [3]. The midgut, which hosts a dense and diverse microbial community in most insect orders, is the primary site of digestion and absorption [4]. In comparison, few studies to date have investigated the bacterial diversity in the foregut (the region dedicated to food intake, storage, filtering and partial digestion). In Diptera (including flies and mosquitoes) and Lepidoptera (butterflies and moths), the crop is a ventral diverticulum of the oesophagus that serves as primary storage organ for sugars from the nectar before it is transferred into the midgut for digestion [2]. Interestingly, a diverse and rich bacterial community was recently observed in the crop of mosquitoes, raising questions about symbiotic associations occurring in this organ [12,13]. Finally, in the hindgut where the bacterial density is very low for certain insect orders and stronger for others (Figure 1), the absorption is completed and feces are formed.

Insect bacterial microbiota offer a wide range of benefits to their host, ranging from increased fecundity [15], oviposition [16], and longevity [17] to shorter larval development [18]. Associated bacteria also influence many other aspects of insect biology, such as complementing host nutrition [19], facilitating dietary breakdown [20], providing protection against pathogens [21,22], and performing the detoxification of xenobiotics or dietary components [23,24,25,26]. The nature of gut microbiota-host associations appears to be variable among insects. While weevils [27], burying beetles [28], and social insects such as termites [29,30], bees [31], or certain ants [32] harbor specialized gut microbial communities mostly transmitted vertically and representing longstanding microbiota-host interactions, other insects like fruit flies or mosquitoes are mainly colonized by transient microbial communities acquired from the environment [33,34].

While an increasing number of studies on insect-associated microbiota have focused on bacteria, other microbial partners such as fungi have been more neglected [35]. Fungal communities (mycobiota) and more particularly yeasts have been demonstrated to be associated with many insect species [36]. Yeasts, which can dominate the mycobiota of certain insects, establish mostly commensal or symbiotic relationships with their host. Like bacteria, yeasts colonize different tissues, such as cuticle, and some yeast species referred to as yeast-like symbionts (YLS) or endosymbionts are localized in fat body specialized cells (mycetocytes) of certain insect species belonging to the Hemiptera and Coleoptera orders [36]. However, yeasts predominantly colonize the digestive tract where they may act as nutrient providers, digestion facilitators, or protectors against pathogens and toxic compounds [37]. Insects are then highly dependent on their gut microbiota, including yeasts, for their development and survival. Based on the degree of dependence, their association can be classified as obligate (or primary) and facultative (or secondary). If YLS located in the mycetocytes of the planthopper *Nilaparvata lugens* [38] and the aphid *Cerataphis brasiliensis* [39] are primary symbionts, some endosymbiotic yeasts are considered secondary symbionts, as they are associated with bacterial species. For example, *Metschnikowia pimensis* and another unidentified YLS (Hp-YSL) of the planthopper *Hishimonus phycitis* are associated with six bacterial endosymbionts including *Sulcia* and *Nasuia* species [40]. Similarly, in several cicada species (*Meimuna opalifera*, *Graptopsaltria nigrofuscata*, *Cryptotympana facialis*, *Hyalessa maculaticollis*, and *Mogannia minuta*), the primary bacterial endosymbionts *Sulcia* is associated with an YLS phylogenetically related to entomoparasitic *Ophiocordyceps* fungi [41]. This review highlights the diversity of commensal and symbiotic yeast communities associated with insects, as well as their impact on insect life-history traits (development, survival, reproduction), immunity, and behavior. As *Drosophila melanogaster*-yeast interactions have been extensively documented [42,43], this insect species was not included in the present review.

## 2. Diversity of Yeast Communities Associated with Insects and Variation Factors

### 2.1. Yeast Community Composition, Structure and Colonization Pathway

The diversity of yeast communities was mostly studied for insect species with a major impact on humans and their environment such as crop auxiliaries (lacewings) [44,45], pollinators (bees, bumblebees, fruit flies, or floricolous beetles) [46,47,48,49], plant pests (moths, planthoppers, bark beetles) [6,50,51,52] and pathogen vectors (mosquitoes, sandflies) [53,54,55]. Yeast communities associated with insects were identified either from entire insect bodies, which were previously surface-sterilized [51,55] or not [48,49], or from dissected organs [13,50,56] using culture-dependent [49,57,58] and independent approaches [59,60]. Independent cultural approaches usually involved DNA extractions from insect tissues followed by the amplification of taxonomic markers allowing a discrimination at the genus or species level, such as the Internal Transcribed Spacer (ITS) regions and the D1/D2 region of 26S ribosomal DNA. Amplified sequences analyzed using DGGE [38,50], T-RFLP [61], Sanger [62,63], or high-throughput sequencing [55,64] were used to characterize insect associated-yeast communities.

Depending on the insect order, the composition of associated-yeast communities was not equally analyzed for all developmental stages (Table S1). While only larvae were studied for Lepidoptera [50,65], the adult stage was preferentially analyzed for many other insect orders [41,51,55,66,67,68]. However, for some species belonging to several insect groups, such as mosquitoes [53], bark or sap beetles [6,69], and planthoppers [64], all life stages were analyzed and the presence of yeast species was detected at all developmental stages (Table S1). These insect-yeast communities are mainly acquired from the environment [68,70,71,72,73]. For example, mosquito larvae acquire yeast communities mainly from the water of breeding sites, while adults obtain it from water at emergence as well as from sugar (plants or flower nectars) and/or blood meals for females during their entire life span [74]. In Hymenoptera (bees and bumblebees), adults acquire yeasts mainly from the nectar of flowers, while larvae obtain them from the provisions (pollen) supplied by adults [63,75].

While a large proportion of yeasts is acquired from the environment, some species are vertically transmitted from adults to larvae. This is typically the case for endosymbiotic yeasts associated with planthoppers, also called yeast-like symbionts (YLS), which are located in specialized cells within the fat body (i.e., mycetocytes) [57,76]. Transovarial transmission of these YLS to the offspring was demonstrated in the brown planthopper *Nilaparvata lugens* [77]. Some yeasts acquired by adults from flower nectar are transmitted vertically to the offspring in the buff-tailed bumblebee (*Bombus terrestris*). This is the case for *Starmerella bombi*, *Wickerhamiella bombiphila*, *Rhodotorula mucilaginosa*, and *Metschnikowia reukaufii,* which have been detected in the digestive tract of several consecutive generations of bumblebee queens [47]. The prevalence of the yeast *Yarrowia lipolytica* in the gut and anal secretions of adult burying beetles, as well as on carcass surfaces and larvae gut, suggests vertical transmission from parents to offspring via the anal secretions [78]. *Wickerhamomyces anomalus* was identified in the reproductive organs of *Anopheles stephensi* mosquitoes that emerged from water-made larval habitats in which the species was undetected [79]. This observation suggests a potential vertical transmission of this yeast.

Yeasts might represent an important part of insect mycobiota. This is the case for certain mosquito species where yeasts account for 19% to 47% of their associated-fungal communities on average [53,80], and can even reach up to 84% of the fungal community in some populations of *Aedes albopictus* [55]. Insect-associated yeast communities are mainly composed of *Ascomycota* and *Saccharomycotina* (Table S1) [6,46,49,55,81,82], such as in floricolous beetles where *Saccharomycotina* species represent 95% of yeast gut communities [62]. Moreover, associated-yeast communities are dominated by a small number of abundant species (1 to 6 species per individual) which differ according to the insect species. For example, one to five different species among *Torulaspora delbrueckii*, *Pichia membranifaciens*, *Starmerella apicola*, *Pichia kluyveri*, *Starmerella meliponinorum*, and *Starmerella bombicola* dominate yeast communities associated with the stingless bee species *Frieseomelitta varia*, *Scaptotrigona aff. postica*, *Scaptotrigona polysticta*, *Tetragonisca angustula angustula*, *Melipona compressipes manaosensis*, and *Melipona scutellaris* [49]. While populations of the Asian tiger mosquito *Ae. albopictus* are largely dominated by *Aureobasidium pullulans*, *Hyphopichia burtonii*, and *Candida* sp. [55], in *Drosophila suzukii* the predominant species are *Hanseniaspora uvarum*, *Pichia terricola*, *P. kluyveri* and *Metschnikowia pulcherrima* [61]. These abundant yeast species are also the most prevalent ones in insect populations, as they are widespread in more than 90% of individuals [55,61]. Preferential associations seem to be established between insect groups and yeast species (Table S1). While floricolous beetles are preferentially associated with yeasts belonging to the genus *Metschnikowia* [48,62], bark beetles favor the genera *Kuraishia*, *Ogataea* and *Cyberlindnera* [6,73]. Bees and fruit flies are preferentially associated with the genera *Starmerella* [49,63,83] and *Hanseniaspora* [46,61,84], respectively. In terms of internal localization, yeasts are mainly present in the gut [47,53,61,82,85,86,87], fat body (mycetocytes) [56,66,88], crop [47,62], or ventral diverticulum [12,13,84]. However, some yeasts were also detected in other organs such as mycetangia [6,89], ovaries [57,79], Malpighian tubules [54], and hemolymph [90,91].

### 2.2. Factors Influencing Yeast Communities Associated with Insects

As previously mentioned, insects acquire a large part of their yeast communities from their nutrient sources (flowers, fruits, sap, etc.) and/or breeding sites [47,53,58,68,71,92]. The environment is therefore one of the main factors shaping yeast communities associated with insects. A study analyzing the structure of yeast communities associated with several *Drosophila* species worldwide has shown that the insect diet has a greater impact than the host species per se [46]. Similarly, Lachance et al. [84] demonstrated that the composition and structure of yeast communities inhabiting the ventral diverticulum of *Drosophila* species feeding on cactus sap (*Drosophila mojavensis*, *D. mettleri* …) are very different from those feeding on sap or tree fruits (*D. pseudoobscura*, *D. Miranda* …). Yeasts vectored by stingless bees differ in southeastern and northern Neotropical savannas of Brazil, suggesting a strong influence of the visited vegetation [49]. Yeast communities associated with bark and ambrosia beetles were demonstrated to be strongly influenced by environmental factors such as host tree species and seasons [68,73,93].

*Saccharomyces cerevisiae* has been identified in the gut or on the body of several insect species all over the world. However, its prevalence in insects of the same species has been found to vary between locations, even though latent factors responsible for such variations have never been clearly identified [94]. Fungal communities associated with the Asian tiger mosquito (*Ae. albopictus*) were more similar among adult individuals at the site level than among countries, and many yeast genera identified in the nectar of flowering plants were also abundant in mosquito individuals (*Aureobasidium*, *Candida*, *Papilotrema*, *Vishniacozyma*, *Kwoniella*, *Hannaella*) [55]. This suggests that environmental conditions and nectar feeding highly contribute to the acquisition of yeasts by mosquitoes. It has been shown that blood ingestion by female mosquitoes of the *Aedes* species, which is often associated with oxidative stress and immune system stimulation, induces a reduction of fungal diversity in the midgut by favoring the development of a few species such as yeasts *Meyerozyma* spp. [95]. The nutritional quality of the plant consumed by the insect can also affect their associated yeast communities. A decrease in the abundance of the yeast-like symbiont (YLS) in the brown planthopper (*N. lugens*) was observed when those pests feed on resistant rice varieties [96]. It has also been shown for the planthopper *Delphacodes kuscheli* that females feeding on nutrient-rich ligular zone of oat plants harbored a higher density of YLS than relatives feeding on the less nutritious leaf apex [97].

Yeasts might exhibit specific tissue tropism or differential tissue tropism as they do not evenly colonize all insect organs. Some of them are localized in specific organs. This is particularly true for YLS, in which tropism is restricted to mycetocytes of certain species of Coleoptera (anobiid beetles) and Hemiptera (planthoppers, aphids, cicadas) [39,41,57,76]. These peculiar cells of the fat body are bigger than other insect cells, present a cytoplasm cluttered by symbiotic microorganisms and are often grouped into an organ called the mycetome. These symbiotic microorganisms supply the insect host with essential nutrients (such as vitamins, amino acids, and so on) [98]. If yeasts preferentially colonize the gut, their density vary depending on their localization. For example, in the green lacewing *Chrysoperla rufilabris*, yeast abundance is higher in the diverticulum (3.7 × 10^3^ CFUs for colony forming units) and foregut (1.6 × 10^3^ CFUs) than in the midgut (2.0 × 10^2^ CFUs) and hindgut (8.3 × 10^1^ CFUs) [99]. In *Ae. albopictus*, yeasts belonging to the *Malassezia* genus are 3 to 55 times more abundant in the ventral diverticulum than in the midgut [13].

Successive molts during insect development lead to the elimination of certain tissues and to the enhancement of the immune system, which strongly impacts yeast communities. Additionally, diet and habitat (aquatic vs. terrestrial) changes during the insect life cycle lead to the acquisition of different yeast species. This is the case for mosquitoes, for which structure and abundance of fungal communities vary across their development with a significant reduction of fungal diversity in newly emerged adults as the midgut undergoes a partial sterilization during metamorphosis from pupae to adult [58,100]. Variations in the abundance of the YLS *W. anomalus* was observed during the life cycle of the planthopper *Laodelphax striatellus*. Indeed, the number of *W. anomalus* gradually increases with the increase of nymphal instar until the 5th instar, and then decreases significantly in the 5th instar, before re-increasing rapidly in the newly-emerged female adult [88].

The sex and social status of insects may also have a significant impact on the structure of yeast communities. In the planthoppers *N. lugens* [77] and *D. kuscheli* [76], YLS abundance gradually increases until the adult stage and remains relatively stable in females, while it strongly decreases upon emergence in males. In *Ae. albopictus*, yeasts belonging to the genus *Aureobasidium* are 11 to 15 times more abundant in the ventral diverticulum and midgut of males compared to females [13]. Yeast community composition is also affected by the social status of their hosts, as has been demonstrated for *Apis mellifera* bees. The gut of young bees and nurses presents a low yeast diversity and is highly dominated by *Saccharomyces* species (representing 97% to 99% of the yeast diversity). In contrast, foraging bees and queens are colonized by diverse yeast species and dominated by *Zygosaccharomyces* species (87%), respectively [86].

## 3. Influence of Yeasts on Insect Life-History Traits and Immune System

### 3.1. Impact on Development, Survival and Reproduction

Whatever their stage of development, insects may use obligate or facultative yeast symbionts to compensate diverse metabolic functions. Yeasts associated with insects are known to facilitate the host feeding on recalcitrant food [82,89,101], provide immunity and protection against various pathogens and parasites [47,102], mediate inter- and intra-specific communication diet [103,104], aid digestion, and supply essential amino acids, metabolic compounds, and nutrients [39,78,105]. Those yeasts are essential for the optimal development and survival of many insects, demonstrated by the fact that *Drosophila suzukii* larvae reared in a yeast-free environment do not reach the pupal stage [106,107]. It has also been demonstrated that axenic mosquito larvae (microbiota-free larvae) exhibit delays in growth of more than six days [18] compared to conventionally-raised ones, or do not develop beyond the first instar, while the development is restored when living yeasts are supplied [108]. Similarly, in the brown planthopper (*N. lugens*), the absence of yeast-like symbionts in mycetocytes prevents the abdominal segmentation and the differentiation of the embryo [109], while a decrease in their density leads to a reduction in nymph weight [110].

Associated yeasts provide dietary supplementation essential for the insect development thanks to their ability to produce essential amino acids, vitamins, proteins, and sterols. Insect pupation requires ecdysteroid hormones, and as insects are not able to synthesize sterols, they must obtain steroids from their diet. Recently, it has been demonstrated that *Zygosaccharomyces* yeasts provide steroid precursors that are essential for pupation to the stingless bee *Scaptotrigona depilis* [111]. Cholesterol is also an essential component of cell membranes and a component of signal transduction pathways. While phytophagous insects typically obtain phytosterols from their host plants, the planthopper *N. lugens* acquires sterols from its yeast-like symbiotes [112]. Yeasts constitute an important source of proteins for the Mediterranean fruit fly (*Ceratitis capitata*) and increase the longevity of laboratory populations [113]. It has been shown that diets with very low yeast proportions led to less protein accumulation in the Medfly larvae [114] and that glutamine enriched yeasts promoted higher pupal recovery and weight [115]. *Saccharomyces cerevisiae* and *Pseudozyma* sp. were reported as the yeast diet with the highest amounts of proteins and carbohydrates leading to the accumulation of energy reserves (proteins, glycogen, lipids) and the development of *Aedes aegypti* larvae (95% to 100% of larvae reach the pupal stage and 85% to 100% the adult stage) [116]. As larvae need to reach a critical mass before moving to the next instar and accomplishing their metamorphosis into an adult, such energy gathering is essential [117].

However, survival and development-time variations were observed depending on yeasts used as diet resources [58]. For example, if *Metschnikowia bicuspidata* and *W. anomalus* promote survival (70% to 80%) and development of *Culex pipiens* larvae (10–15% of larvae achieving their pupal stage), *Cryptococcus gattii* impacts negatively on pupation (no pupae observed) and larval survival (less than 30%) [58]. Concerning the fruit fly *D. suzukii*, larvae fed *S. cerevisiae* develop faster (11 days) than those reared in the presence of *H. uvarum* (14 days) or *P. terricola* (18 days). *Candida* sp., *H. uvarum*, and *S. cerevisiae* confer a better survival rate to larvae (38% to 51%) than *M. pulcherrima*, *R. mucilaginosa* and *P. terricola* (4% to 19%) [106,107]. Differences were also observed for adults, since fruit flies fed *S. cerevisiae* have longer thoraxes and larger wings [107]. The presence of yeasts in the diet of the bumblebee *B. terrestris* also promotes colony development in terms of the number of pupae and workers [118]. However, such observation is species-dependent as *Wickerhamiella bombiphila*, *Metschnikowia gruessii* and *R. mucilaginosa* induced the strongest positive impact on the development of these bumblebee colonies [118]. Similarly, biomass of the red fire ant colonies with yeasts was significantly greater than ant colonies without yeasts during winter and spring months [90]. A study focusing on the codling moth (*Cydia pomonella*) showed that fruits colonized by *Metschnikowia andauensis* led to a decrease of 25% in larvae mortality and an increase of 55% in the number of pupae after 35 days of development [65]. This decrease in mortality is partly explained by the yeast presence, which reduces the fruit colonization by molds by 35% [65].

Adult burying beetles *Nicrophorus vespilloides* discover and bury carcasses, lay eggs in nearby soil, and the hatching larvae migrate to feed on the carcass. Preservation of the carcass during the breeding cycle is thought to be achieved by smearing the carcass with anal and oral secretions. The abundance of *Yarrowia* species, which have broad spectrum antimicrobial activity, could offer a mechanism to prevent carcass colonization by undesirable bacteria and fungi [78]. Moreover, these yeast symbionts are thought to be involved in the digestion of the carcass and in the supply of essential nutrients to their host. The functional analysis of *Yarrowia* transcripts revealed their potential to secrete a large number of proteases and lipases. Their role in sterol production in the rectum is supported by the finding that both sterol modifying enzymes (sterol reductases) and sterol transport proteins (lipophorins) are expressed at high levels, specifically in the host rectum [78]. For insects feeding on recalcitrant substrate such as wood, yeast symbionts participate to nutrient acquisition and detoxification of defensive plant compounds [6,73]. Some yeasts such as *Cyberlindnera americana* and *Ogataea pini* are able to utilize terpenoids as carbon sources, the primary defensive chemicals constitutively present in the phloem resins of conifers, which may be important for *Dendroctonus* and *Ips* beetle tolerance of defensive phytotoxins [6,101]. Several structural carbohydrates of host plants (e.g., cellulose, hemicelluloses) are not easily degraded by insects. Some of these compounds are partially hydrolyzed by digestive enzymes produced by yeast symbionts present in their gut. *Candida pseudorhagii*, the most frequently occurred yeast in *Reticulitermes chinensis* termite guts, showed a strong xylanolytic activity and a high D-xylose fermentation capacity [82]. *Saccharomycopsis* sp. and *Cyberlindnera* sp. associated with the ship timber beetle *Elateroides flabellicornis* are able to assimilate cellobiose [89]. Insects such as grasshoppers feed on leaves which are rich in cellulose. Since these insects synthetize few cellulases, this polymer of glucose is partially hydrolyzed in their midgut by the microbiota, and particularly certain *Basidiomycota* yeasts harboring high cellulolytic activities such as *Papiliotrema* and *Saitozyma* species [67].

At the adult stage, several phytophagous and blood-sucking insects feed on plant substances enriched in fructose, glucose, and sucrose [111,112,116]. If a certain proportion of these plant sugars is digested by enzymes contained in saliva and directly assimilated by the insect, most of them are stored in the crop or in the ventral diverticulum where a wide variety of yeast genera are present, such as *Candida*, *Debaryomyces*, *Hanseniaspora*, *Meyerozyma*, *Metschnikowia*, and *Pichia* (Table S1) [12,13,47,84,119]. Sugars will then be gradually transported to the midgut where they will preferably be used as an energy source by the microbiota, and particularly yeasts [87]. For example, it has been shown that yeasts of the genus *Malassezia* associated with both male and female *Ae. albopictus* actively utilize fructose, while yeasts of the genus *Cyberlindnera* are more active in females [87]. 

Beside their nutritional role, yeasts are also involved in the induction of gut hypoxia functions in insects. It was demonstrated that *S. cerevisiae* induces hypoxia, serving as a signal for growth and molting, in the gut of *Ae. aegypti* [108]. By supplying Riboflavin, like bacteria, yeasts should stimulate the biosynthesis of flavin adenine dinucleotide (FAD) and flavin mononucleotide (FMN) in mosquito’s cells. The production of these two cofactors essential for the functioning of enzymes involved in the respiratory metabolism might stimulate the respiration of mosquito’s intestinal cells and reduce gut oxygen levels below 5% [120]. This gut hypoxia activates hypoxia-induced transcription factors (HIFs) that stimulate signal transduction cascade leading to the accumulation of neutral lipids in the fat body and molting [108]. Neutral lipids, which are steroid precursors, are essential for molting and pupation as they are required for the synthesis of ecdysteroid hormones [117]. Such mechanisms could likely be extended to further mosquito species as the presence of *S. cerevisiae* also promoted the development of axenic *Cx. pipiens* larvae [100]. In *Ae. aegypti* adult mosquitoes, when the microbiota was modified to be enriched or exclusively composed by yeasts, individuals were found to maintain a high percentage of survival (68–100%) [108,116]. Similar results were observed for the adults of the planthopper species *Sogatella furcifera* [121] and *N. lugens* [122] since a fungicide treatment, which reduces YLS density in mycetocytes, decreases the insect survival by 60%. Yeasts can also have an impact on insect reproduction. For example, the ingestion by *D. suzukii* adult females of *H. uvarum*, *Saccharomycopsis vini* and *Candida* sp. promotes their survival and their fertility (number of eggs laid) [106,123]. Conversely, concerning the bumblebee *Bombus impatiens*, the presence of yeasts such as *M. reukaufii* does not affect the number of eggs laid by females [124].

Uric acid is a nitrogenous waste substance produced either during the purine metabolism or blood digestion. It is usually accumulated within the Malpighi tubes before being excreted. In the sand fly *Phlebotomus perniciosus*, the yeast *M. guilliermondii* colonizes the distal part of female Malpighian tubules. Moreover, *M. guilliermondii* possesses an uricolytic activity and presents in its genome the complete uric acid degradation pathway, suggesting that this yeast might contribute to the removal of the excess of uric acid after the blood meal of the insect host [54]. In the planthopper *N. lugens*, which does not excrete uric acid nor present its own uricase activity, it was demonstrated that yeast-like symbiotes use and recycle this nitrogenous waste [125]. The absence of YSL was systematically associated with a high accumulation of uric acid and an absence of uricase activity in the insect tissues [125]. Similarly, beside their nutritional role, yeast-like symbiotes associated with the Asian mealybug (*Kerria lacca*) are involved in the detoxification of plant self-defense chemicals such as resins or latex [126]. 

### 3.2. Impact on Insect Immune Response and Resistance against Infections

**Figure 2:**
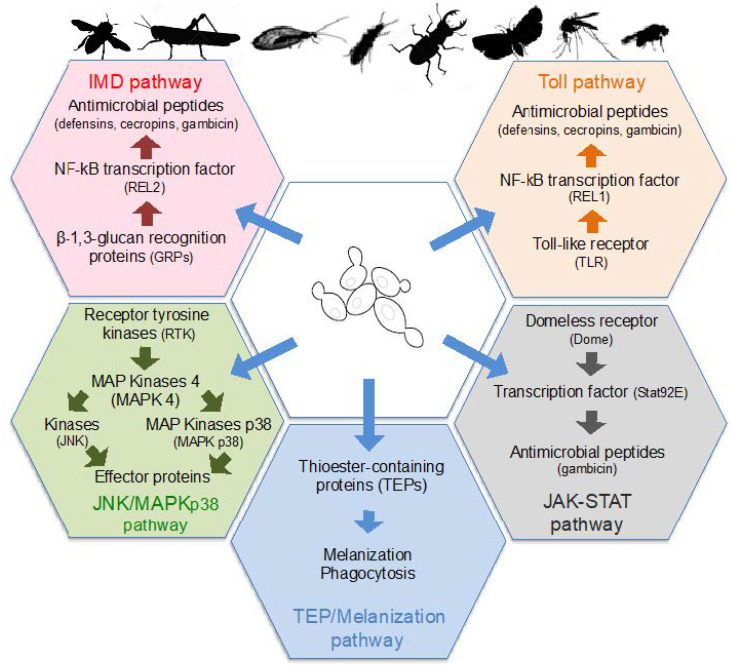
Signaling pathways of insects’ innate immunity stimulated by yeast colonization. Yeast surface molecules or secondary metabolites are recognized by specific receptors. This recognition induces the activation of kinases or transcription factors that stimulate the production of antimicrobial peptides or other effector proteins, as well as phagocytosis of yeast cells and melanization. These signaling pathways stimulated by yeast are Toll, Imd (Immune deficiency), JAK/STAT (Janus Kinase/Signal Transducer), JNK/MAPKp38 (Jun N-terminal Kinase/Mitogen Activated Protein Kinase p38), TEP (ThioEster-containing Protein), and TEP/Melanization.

Insects only have an innate immune system that is based on the recognition of conserved microbe-associated molecular patterns (MAMPs) by a set of pattern-recognition receptors (PRRs) localized on the surface of host cells [127]. Several classes of PRRs are able to detect fungal surface molecules and secondary metabolites, which then induce the activation of protein kinases or transcription factors. In turn, those protein kinases and transcription factors stimulate the production of insect antimicrobial peptides (AMPs) including cecropins, defensins, diptericin, and gambicin, or other effector molecules, as well as phagocytic and melanization responses (Figure 2). Infection by fungi, and therefore yeasts, activate several signaling pathways, and more particularly the Toll and TEP/Melanization pathways [127,128].

Yeast species not naturally present in insect tissues are considered to be pathogens and their entrance activates the immune system [129]. For instance, the injection of *Saccharomyces cerevisiae* and *Candida albicans* in the hemolymph of the mosquito species *Anopheles albimanus* and *Culex quinquefasciatus* induces melanization of fungal cells after their recognition by thioester-containing proteins (TEPs). Fungal cells die following nutrient deprivation but are not phagocytosed by hemocytes [91,130]. In the diamondback moth *Plutella xylostella*, oral infection with the yeast *Komagataella pastoris* activates the expression of 24 insect immunity-related genes by inducing the overexpression of proteins involved in the recognition of the β-1,3-glucan, a fungal wall compound [131]. However, gut-inhabiting yeasts also modulate the insect immune response in order to maintain and develop in the insect gut. In *A. mellifera* bees, the yeast *W. anomalus* could induce or repress the expression of some genes involved in innate immunity [132]. Moreover, by stimulating the immune system, yeasts can also prevent host colonization by other microorganisms such as pathogens (entomopathogenic microorganisms or human pathogens) and thus interfere with insect vector competence (mosquitoes, sandflies). As examples, strains of *W. anomalus* unable to produce toxins reduce by 38% the infection of *An. stephensi* by *Plasmodium berghei* (protozoan responsible for malaria in humans) probably by stimulating the immune system [133]. In addition, the presence of *S. cerevisiae* is able to stimulate the immune system of the European paper wasp *Polistes dominula* leading to a faster and efficient removal of the bacterium *Escherichia coli* [134].

Other mechanisms, such as resource competition or production of antimicrobial compounds (toxins or other), allow yeasts to inhibit colonization of the insect host by entomopathogens or human pathogens. An in vitro study has demonstrated that yeasts of the species *M. reukaufii*, *S. bombi*, *W. bombiphila*, previously isolated from the midgut of the bumblebee *B. terrestris* and known to be competitive for resource consumption reduce the development of the natural parasite of this insect (the protozoan *Crithidia bombi*) by 25% to 85% [118]. 

Regarding the impact of yeasts on insect vector competence, the only known examples concern the yeast *W. anomalus* and the protozoan *P. berghei*, the malaria parasite transmitted by *Anopheles* mosquitoes and in particular *An. stephensi*. It has been demonstrated that some strains of *W. anomalus*, naturally present in the midgut of *An. stephensi* [79], could produce lethal toxins with a broad spectrum of antifungal and antiparasitic activities [133]. Valzano et al. [135] have also shown that these mechanisms of inhibition are partly based on the β-1,3-glucanase activity of these toxins. Thus, due to their presence in the midgut and the glucanase activity of their toxins, *W. anomalus* yeasts inhibit the development of *P. berghei* in female *Anopheles* by causing the death of the parasites through an extensive damage of their cell-walls rich in glucans. Quantitatively, toxin-producing strains reduce the number of parasites (zygotes and ookinetes) in female *Anopheles* by 65% [102]. In contrast to in vitro studies, where a 90% decrease in oocysts and sporozoites has been observed [135], the lack of antiparasitic effect in vivo on these two forms of the sporogonic phase could be explained by their localization outside the lumen of the midgut, and therefore the absence of contact with toxins [102].

## 4. Impact of Yeasts and Their Volatile Compounds on Insect Behavior

### 4.1. Influence on Feeding Behavior

**Figure 3:**
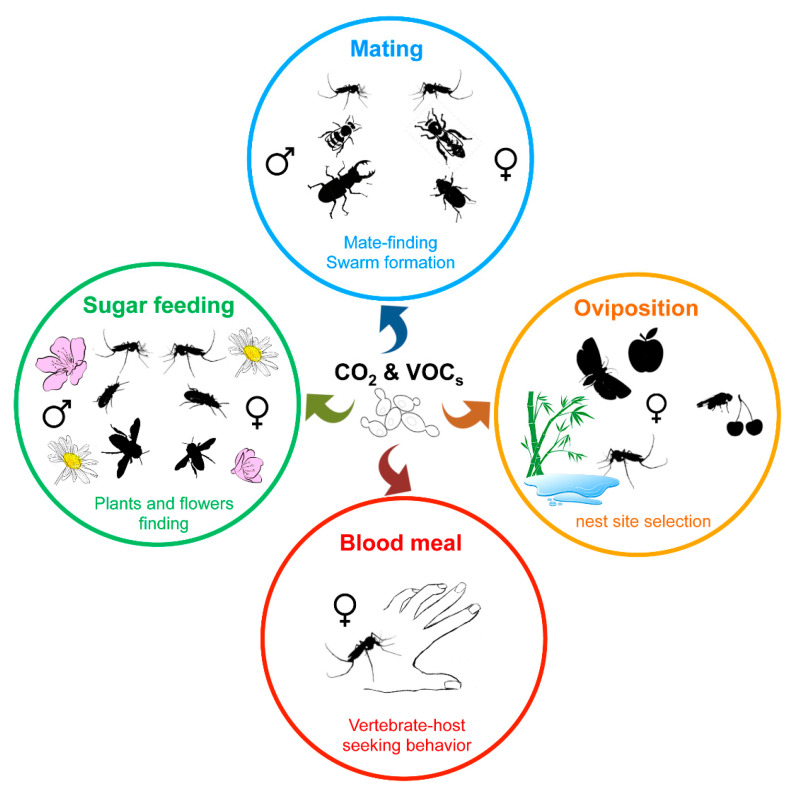
Influence of yeast volatile compounds on blood-sucking and phytophagous insect behavior. Insects use olfactory perception of chemical cues, such as CO_2_ or volatile organic compounds (VOCs), to find favorable nest sites for larval development, vertebrate hosts, flowering plants, or mating partners.

Besides visual signals, insects largely use the olfactory perception of chemical signals, such as emissions of CO_2_ and pheromones or volatile organic compounds (VOCs), to move toward or find a partner, a food source (nectar, blood, etc.) or a nest site (Figure 3) [104,136,137,138,139]. While plants, vertebrate hosts, or insects themselves directly produce such chemical compounds, environmental microorganisms or insect microbiota also contribute to the release of such kairomones. Indeed, CO_2_ as along with a wide variety of volatile secondary metabolites are emitted by yeasts as by-products of fermentation, and play a role in insect attraction [104,140]. 

The ability to synthesize and release volatile compounds is also an old phenotypic trait that has been preserved in yeasts [141]. Several studies have shown that the simultaneous presence of VOCs and CO_2_ both produced by yeasts during the fermentation of various carbon sources is more effective to attract insects than inert yeasts, industrial CO_2_, or octenol (aromatic compound of plant or fungal origin widely used in commercial traps to capture biting insects) used alone [142,143,144,145]. For example, it was recently shown that the yeast *Cyberlindnera jadinii* adult attracted more efficiently green lacewing adults (*Chrysoperla comanche*) when it was alive, thus demonstrating the importance of the volatile compounds emitted by yeasts to attract these insects [146].

The presence of yeasts in the nectar strongly impacts the search for food of flower-visiting insects [104]. Studies in this area have mainly focused on the most common insect pollinators (bees, bumblebees). By emitting large quantities of ethanol, 2-methylbutan-1-ol, and to a lesser extent 2-methylpropan-1-ol, 2-phenylethanol and ethyl acetate, the nectariferous yeast *M. reukaufii* strongly impacts the behavior of the bumblebee species *Bombus friseanus* [147] and *B. impatiens* [124,136]. These species preferentially forage and spend a longer time (34% extended residence time) on plant species with flowers harboring this yeast, thus improving seed production by 10% [124,136,147]. Conversely, other yeast species commonly found in nectar, such as *W. bombiphila*, *M. gruessii*, or *R. mucilaginosa*, do not seem to have a significant influence on the foraging of the bumblebee *B. terrestris*. However, they stimulate nest size (number of individuals) by decreasing the *C. bombi* infection risk [118].

Blood-sucking insects such as mosquitoes which feed on both nectar (males, females) and blood (gravid females require blood meals to complete oogenesis), locate their food sources through volatile compounds (CO_2_ and VOCs) partly emitted by yeasts found in plant nectar and on the skin of vertebrate hosts [138]. However, unlike nectar-living yeasts, the attractiveness of the yeasts found on human or vertebrate skins has never been tested. Depending on the nature of the VOCs generated and their concentration, attraction and repulsion behaviors have been observed towards mosquitoes [148]. Even if the fermentation by yeasts of complex carbohydrates such as honey generates a greater production of VOCs, including attractant compounds such as hexanoic acid or phenylethyl alcohol, sucrose attracts a greater number of mosquitoes. In this case, the absence of certain VOCs with repulsive properties could promote the attraction of mosquitoes [148]. In addition to their impact on the behavior of adult mosquitoes, yeasts also impact the feeding behavior of larvae. Yeasts that promote the development of larvae, through the supply of nutrients or the accumulation of reserves following the detection of a gut hypoxic signal [108,116], attract and strongly impact the behavior of larvae [149,150]. Indeed, the presence of *S. cerevisiae* in the larval food of *Anopheles gambiae* reduces the average velocity, rotations, and number of movements of larvae, while increasing their resting time [150].

A recent study has demonstrated that yeasts isolated from flowers, leaves, or fruits emitted specific VOC profiles that influence the feeding behavior of larvae of the moth *Spodoptera littoralis*. These larvae feed exclusively on leaves and are strongly attracted by yeasts retrieved from the plant phyllosphere (*Metschnikowia lopburiensis* and *Papiliotrema nemorosus*), while most of the yeasts isolated from fruits (*M. andauensis* and *M. pulcherrima*) are repellent. The attractive VOCs emitted specifically by the yeasts of the plant phyllosphere are geranyl acetone, cyclohexanone, 2-thyl-1-benzofuran, and 1,3,5-undecatriene [151].

### 4.2. Influence on Nest Site and Partner Choice

Mate choice and species recognition can be strongly influenced by the presence of yeasts and the release of their VOCs. In the *Drosophila* genus, the reproductive success partly relies on the size of the males. It was shown that at equal size the females favor the males whose heads are covered with yeasts. Moreover, during courtship displays, males regurgitate a nutrient liquid containing yeasts, which attracts females [61]. Similarly, males of the species *C. comanche* produce yeast-laden regurgitant composed by the genus *Metschnikowia* that attracts females [146]. Mate choice and recognition are based above all on the detection of volatile and/or contact (cuticular hydrocarbons) sex pheromones produced by the insect or its associated microbiota [152]. While the production of pheromones by microorganisms has only been shown for a few bacteria, it was shown that an alteration in the microbiota composition (including yeasts) is associated with a decrease in insect reproductive success [152].

Yeasts play an important role in the choice of a nest site, regardless of the insect. In mosquitoes, gravid females assess the acceptability of breeding sites using chemical signals from larvae, eggs, and/or the microbial community present in the aquatic larval habitat [138]. *Ae. aegypti* gravid females tend to promote breeding sites containing eggs and larvae of the same species. The presence in the water of breeding sites of *Candida pseudoglaebosa* (yeast species that naturally colonizes the midgut of *Ae. aegypti* mosquitoes) attracts gravid females and promotes egg laying [153]. Conversely *S. cerevisiae*, which does not belong to the mosquito mycobiota, does not seem to attract gravid females in *Cx. pipiens* [100]. The beetle *Araecerus fasciculatus*, which lays its eggs in coffee beans, would be able to locate its host plants using VOCs (2-phenylethanol and 2-phenylethyl acetate) released by certain yeasts [154].

Larvae of the insect pests *Rhagoletis batava*, *D. suzukii*, and *C. pomonella* develop inside the fruits that are still attached to the tree [65,106,137]. As previously observed, gravid females tend to favor niches (fruits) containing eggs and larvae of the same species [65,155]. In general, the choice of a nest site appears to be strongly guided by the presence of yeasts able to colonize larval guts and promote their development. These yeasts emit many volatile compounds (ketones, phenols, terpenes, esters, alcohols, fatty acids, etc.) that stimulate and attract gravid females [65,137]. Thus, yeasts of the genus *Metschnikowia* (*M. andauensis* and *M. pulcherrima*) living in apples attract gravid *C. pomonella* females through the release of volatile compounds, and promote egg-laying [65]. Likewise, in the fruit fly *D. suzukii*, the yeast species *S. cerevisiae* and *Candida* sp. inoculated in cherries promote egg-laying by attracting gravid females [106]. Finally, it was suggested that some yeasts naturally associated with *D. suzukii* and fruits such as *H. uvarum*, would be able to influence more post-mating eating behavior rather than the choice of nest site [156].

The bark beetle *Dendroctonus ponderosae* appears to rely primarily on microbial symbionts for terminating aggregation and mass attack on individual host trees. Indeed, Hunt and Borden [157] demonstrated that two isolated yeasts, *Kusarishia capsulata* and *Ogataea pini*, were able to metabolically convert cis- and trans-verbenol into verbenone. While cis- and trans-verbenol are *D. ponderosae* aggregation pheromones, verbenone acts as an anti-aggregation pheromone. They surmised that high levels of colonization by yeasts in host trees are signaling that the substrate may no longer be suitable for reproduction.

## 5. Conclusions

Despite a growing number of studies on the impact of yeasts on the biology and behavior of insects, these are still very limited and mainly concern the few insect species closely associated with agricultural systems and ecosystem services (e.g., bees, planthoppers, fruit flies). The rules governing these interactions and their effects on microbial and animal lives are far from completely understood, and depicting relations between yeasts and insects will represent a fundamental step towards a better understanding of ecological and evolutionary interactions. By describing the yeast populations associated with a wider range of insects, it will eventually be possible to assess species-specific interactions. Physiology analyses of yeasts found in these environments will further expand our knowledge in terms of insect-benefits. The benefits gained by yeasts from their association with insects have been little investigated and are poorly understood. While our current knowledge recognizes the importance of insects for the dispersion of yeasts to new substrates or habitats, the benefits of this association for yeast may be more diverse. Indeed, recently, it was suggested that in the absence of flowers and fruits during the winter period, the yeast *M. reukaufii* survived in the bumblebee gut and recolonized flowers in spring after the end of their hosts’ hibernation [47]. Stefanini et al. [158] demonstrated that the gut of wasps favored intra- and interspecific mating of *Saccharomyces* strains, thus supporting the hypothesis that this environment might promote the emergence of new yeast strains. Finally, the study of VOCs produced by yeast is also a promising field of research, as many of them can attract pest insects and could therefore be used in attract-and-kill or monitoring traps for pest management.

## Supplementary Materials

The following are available online at https://www.mdpi.com/article/10.3390/microorganisms9081552/s1, Table S1: Overview of the yeast species detected in insects according to their stage of development [6,13,26,30,39,43,46,47,48,49,50,52,53,54,55,60,61,62,64,65,66,67,68,71,72,74,77,78,79,83,84,85,86,87,88,89,91,92,94,98,104,120,157,158,159,160,161,162,163,164,165,166,167,168,169,170,171,172,173,174,175,176,177,178,179,180].
